# Ultrasound-triggered chitosan interfacial crosslinking for core–shell nanoemulsions with enhanced salidroside bioaccessibility

**DOI:** 10.1016/j.fochx.2026.104384

**Published:** 2026-08-28

**Authors:** Liang Dong, Guicheng Liu, Xin Zhang, Xiaojin Zhang, Sijing Liu, Shuqi Niu, Degui Chang, Xujun Yu, Jinlin Guo

**Affiliations:** a School of Medical and Life Sciences, Chengdu University of Traditional Chinese Medicine, Chengdu 611137, China; b TCM Regulating Metabolic Diseases Key Laboratory of Sichuan Province, Hospital of Chengdu University of Traditional Chinese Medicine, Chengdu 610075, China; c Chengdu University of Traditional Chinese Medicine, Chengdu 611137, China; d College of Medical Technology, Chengdu University of Traditional Chinese Medicine, Chengdu 611137, China; e The Second Affiliated Hospital of Chengdu University of Traditional Chinese Medicine, Chengdu 611137, China; f State Key Laboratory of Southwestern Chinese Medicine Resources, College of Pharmacy, Chengdu University of Traditional Chinese Medicine, Chengdu 611137, China

**Keywords:** Nanoemulsion, Chitosan, Ultrasonic, Core-shell structure, Salidroside

## Abstract

An ultrasound-driven strategy developed to construct chitosan–tripolyphosphate ionic crosslinked shells of lecithin-stabilized nanoemulsions, enhancing salidroside encapsulation. Ultrasound accelerated phosphate mass transfer toward chitosan-adsorbed layers, homogenizing crosslinking density, increasing the degree of crosslinking to 0.78 and yielding a compact interfacial gel network with the highest encapsulation efficiency of 69.21%. Nanoemulsion transformed from a viscous fluid into viscoelastic weak hydrocolloids with narrowed relaxation time distribution. Coupled hydrogen bonding and ionic interactions between phosphate and chitosan hydroxyl/amino groups formed, while ^1^H NMR revealed restricted oil molecular mobility and C_2_—H downfield shifting indicative of –NH_3_^+^/phosphate ion-pair formation. In vitro simulated digestion demonstrated high salidroside bioaccessibility (25%) and facilitated mixed micelle formation, while post-digestion micelles significantly attenuated hypoxia-induced oxidative damage in RAW264.7 macrophages, reducing ROS/NO levels, restoring ATP production, and downregulating HIF-1α signaling. These findings establish ultrasound-driven interfacial ionic crosslinking as an effective approach for engineering robust core–shell nanoemulsions to enhancing bioactivity of food ingredients.

## Highlights

• Ultrasound promotes chitosan–phosphate interfacial crosslinking at emulsion droplets;
• Ultrasound enhances ionic and hydrogen bonding between chitosan and phosphate;
• Highly crosslinked core–shell structure optimizes salidroside sustained release;
• Assembled gelled core–shell layer enhances environmental stability of emulsions;
• Core–shell emulsions enhance salidroside bioaccessibility and anti-hypoxic efficacy;

## 1 Introduction

Salidroside, a bioactive compound distinguished by exceptional antioxidant, anti-inflammatory, and mitochondrial protective properties, has been demonstrated to stabilize hypoxia-inducible factor-1α (HIF-1α) and upregulate vascular endothelial growth factor expression, thereby enhancing cellular resilience under hypoxic conditions (Liu et al., 2024). However, its clinical translation is severely hampered by unfavorable pharmacokinetic characteristics. The pronounced hydrophilicity of salidroside impedes effective traversal of the lipid bilayer barriers of cell membranes, while its vulnerability to pH fluctuations and enzymatic hydrolysis within the gastrointestinal tract leads to extremely poor oral bioavailability (Du et al., 2022). To circumvent these delivery barriers, the rational design of efficient nano-delivery systems represents a pivotal strategy for enhancing salidroside bioavailability. Nanoemulsions, as multiphase colloidal dispersions, offer excellent biocompatibility and robust solubilization capacity for bioactive compounds, rendering them promising carrier platforms for oral nutrients delivery (Zhang et al., 2024). Nevertheless, conventional emulsion systems frequently exhibit structural fragility when confronted with the aggressive physicochemical environment of the gastrointestinal(GI) tract (Niu et al., 2023). The displacement of emulsifiers by bile salts, enzymatic hydrolysis of phospholipids by pancreatic lipase, and drastic ionic strength shifts can readily compromise interfacial integrity, triggering droplet coalescence and premature nutrients release before the active payload reaches the intestinal epithelium (Lee et al., 2024).

Chitosan, a cationic polysaccharide derived from the partial deacetylation of chitin, comprises β-1,4-linked 2-amino-2-deoxy-d-glucose and *N*-acetylglucosamine residues (Yadav et al., 2023). The abundant free amino and hydroxyl functionalities distributed along its backbone endow chitosan with superior biocompatibility, biodegradability, and film-forming capacity (Chen et al., 2023). When deployed at oil-water interfaces in conjunction with amphiphilic emulsifiers such as lecithin, chitosan can electrostatically anchor onto droplet surfaces, reducing interfacial tension and preventing flocculation. Crucially, these amino moieties serve as reactive sites for ionic crosslinking with multivalent anions, enabling the formation of a dense three-dimensional polymeric network shell. Such interfacial self-assembly yields a core-shell architecture that markedly reinforces droplet mechanical robustness, preserving structural integrity against shear forces, thermal fluctuations, and ionic perturbations, while concurrently retarding the diffusion of encapsulated actives and shielding them from digestive fluid erosion (Wu et al., 2023). However, conventional static interfacial crosslinking is inherently constrained by the heterogeneous diffusion of crosslinking agents at curved droplet interfaces, frequently resulting in non-uniform shell thickness and suboptimal mechanical stability.

Ultrasound, as a non-thermal physical processing modality, generates intense acoustic cavitation characterized by localized microjetting, turbulent microstreaming, and transient hotspots (Zhou et al., 2022). These cavitation-mediated phenomena can substantially accelerate the mass transfer of crosslinking agents toward chitosan-adsorbed interfacial layers, facilitating the homogeneous densification of ionic crosslinking networks (Jadhav et al., 2024). Although ultrasound-assisted interfacial crosslinking has been exploited to enhance the structural stability of microcapsules and nanoparticles, its application in constructing chitosan-based core-shell nanoemulsions for improving the oral delivery efficiency of salidroside remains unexplored.

In the present work, lecithin-stabilized nanoemulsions were employed as the foundational carrier, and an ultrasound-driven strategy was implemented to promote the formation of a compact chitosan-phosphate crosslinked shell at the droplet interface for salidroside encapsulation. The physicochemical properties of the resultant core-shell nanoemulsions were systematically characterized, and the underlying ionic crosslinking mechanism at the oil-water interface was mechanistically elucidated. Through in vitro simulated gastrointestinal digestion assays, the dynamic evolution of droplet dimensions, zeta potential, and microstructural morphology was monitored to assess GI stability and nutrients release profiles. Furthermore, the micellar phase recovered post-digestion was isolated and applied to a hypoxia-injured RAW264.7 macrophage model to evaluate the cytoprotective efficacy of the delivered salidroside. This study aims to provide theoretical insights and technical guidance for the rational design of ultrasound-engineered core-shell nanoemulsions capable of enhancing the oral bioavailability of salidroside.

## 2 Materials and methods

### 2.1 Materials

Salidroside and lecithin were purchased from Yuanye Bio-technology Co., Ltd. (Shanghai, China). Nile Red, fluorescein isothiocyanate (FITC), pepsin (3256 U/mg), and pancreatin (1587 U/mg) were purchased from Sigma-Aldrich (St. Louis, MO, USA). Bile salts (porcine), Propidium iodide (PI), 2′,7′-dichlorodihydrofluorescein diacetate (DCFH-DA), Calcein-AM, and 3-amino,4-aminomethyl-2′,7′-difluorofluorescein diacetate (DAF-FM DA) were obtained from Macklin Biochemical Co., Ltd. (Shanghai, China).

### 2.2 Preparation of nanoemulsion

MCT was used as the oil phase at a proportion of 20%; salidroside was dissolved in 100 mM pH 7.0 phosphate buffer at a concentration of 3 mg/mL, and 2% lecithin was added as a stabilizer. The system was dispersed at 10,000 rpm for 2 min, then was homogenized under 100 MPa pressure and was cycled 3 times (KBS KS-1500 L,Kebais Nano Technology (Shanghai) Co., Ltd., China), and the nanoemulsion was thus prepared. The gel layer nanoemulsion was prepared by dissolving chitosan (1%, *w*/*v*) in acetic acid solution (pH 5.0). The nanoemulsion system was added to the chitosan solution, and the mixture was slowly stirred at 600 rpm for 5 min under a magnetic field. Adjust the pH to 5.0, gradually add the phosphate solution(100 mM pH 7.0), with the total amount of the phosphate solution being 1/10 or 1/5 of the chitosan. Ultrasonic treatment was carried out for 15 min at 300 W, 20 kHz and 30% amplitude (VSX800, Sonics, USA), and these parameters were selected based on the conditions reported by Zhao et al. (2024). Throughout the process, the temperature was maintained below 25 °C in an ice bath, and a 10 mm probe was used to prepare a salidroside-loaded nanoemulsion with a uniform gel layer. The experimental groups were set up as follows: control group, chitosan group (CH), chitosan + low phosphate group (1/10) / high phosphate (1/5) group (L-CH-TSP and H-CH-TSP), chitosan+ low phosphate group (1/10) / high phosphate (1/5) + ultrasound group (L-CH-TSP-UL and H-CH-TSP-UL).

### 2.3 Characterization of the physicochemical properties of nanoemulsion

#### 2.3.1 **Psize and** ζ-**potential**

Droplet dimensions, surface charge, and dispersity were quantified by Dynamic Light Scattering (DLS) and electrophoretic light scattering. Emulsions were diluted 400-fold (size/PDI) or 1000-fold (ζ-potential) with Milli-Q water, equilibrated for 120 s, and analyzed over 12 runs (Zetasizer Nano ZS-90, Malvern, UK).

#### 2.3.2 The crosslinking degree

The nanoemulsion was lyophilized; 20 mg of the lyophilized sample was dispersed in 0.1 M sodium bicarbonate buffer at pH 8.5, followed by the addition of 0.01 M 2,4,6-trinitrobenzenesulfonic acid (TNBS) solution. The mixture was incubated at 37 °C in the dark for 2 h, centrifuged, and the supernatant was collected. The absorbance was measured at 420 nm. The degree of cross-linking was calculated according to the following formula:

$$\mathrm{Degree of crosslinking}\;\left(\%\right)=\frac{A_{\mathrm{control}}-A_{\mathrm{sample}}}{A_{\mathrm{control}}-A_{\mathrm{blank}}}$$

where A_control_, A_sample_, A_blank_, and represent the absorbance of the control group, sample group, and blank group, respectively.

#### 2.3.3 The encapsulation rate and loading capacity

The nanoemulsion sample was transferred into an ultrafiltration centrifuge tube with 10 kDa molecular weight cut-off and centrifuged at 12,000 rpm at 4 °C for 30 min to separate the unencapsulated free salidroside. The filtrate was collected for subsequent analysis. An equal amount of nanoemulsion was mixed with acetonitrile at a volume ratio of 1:3, followed by ultrasonication to disrupt the nanoemulsion. The total salidroside concentration was then determined by HPLC (1260 Infinity II Prime, Agilent, USA) using isocratic elution to calculate the total content. Prior to HPLC analysis, all samples were filtered through a 0.45 μm membrane filter. The chromatographic conditions were as follows: C_18_ BEH column (50 mm × 2.1 mm, 1.7 μm), mobile phase, methanol-water (15:85, *v*/*v*), detection wavelength at 275 nm, flow rate 1.0 mL/min, column temperature 30 °C, and injection volume 10 μL. Prepare a 1.43 mg/mL stock solution of the salidroside standard. When required, prepare solutions of varying concentrations from this stock solution and determine the peak areas under the chromatographic conditions described above.

#### 2.3.4 The release rate of salidroside

The 20 mL salidroside-loaded nanoemulsion was transferred into a dialysis bag with 3.5 kDa molecular weight cut-off, which was then immersed in 300 mL of pH 7.0 0.1 M phosphate buffer solution as the release medium. The dialysis bag was incubated in a thermostatic shaker at 37 °C and 100 rpm. At predetermined time intervals (30, 60, 120, 180, 240, and 360 min), 1 mL of the release medium was withdrawn and immediately replenished with an equal volume of fresh medium to maintain sink conditions. The concentration of salidroside in the release medium was determined by HPLC, and the cumulative release rate was calculated.

#### 2.3.5 The laser scanning confocal microscopy (CLSM) imaging

CLSM imaging was conducted on a Leica TCS SP8 STED 3× (60× objective) to map the oil-chitosan interfacial topology. Fresh emulsions were stained by spiking 20 μL of 1 mg/mL Nile Red/FITC cocktail into 1 mL aliquots. After gentle shaking, 10 μL was mounted on glass slides under coverslips. Nile Red (Ex/Em: 488/590 nm) and FITC (Ex/Em: 488/525 nm) were sequentially excited to avoid spectral bleed-through.

#### 2.3.6 The rheological properties of nanoemulsion

Rheological characterization was performed on an Anton Paar MCR301 rheometer (50 mm parallel plate, 0.1 mm gap). Emulsion samples were loaded and rim-sealed with paraffin oil to prevent evaporation. Frequency sweeps (1–100 rad/s, γ = 2%, 25 °C) within the linear viscoelastic region furnished G′ and G″. Steady-shear tests (0.1–100 s^−1^) provided viscosity profiles. Data were replotted as Cole–Cole (G″ vs. G′) and Han (log G″ vs. log G′) diagrams for relaxation analysis.

### 2.4 Interactions among the components of the nanoemulsion

#### 2.4.1 The Raman spectra

The nanoemulsion was dropped onto a glass slide and scanned by a Raman spectrometer (In Via, Bruker, Germany) equipped with a 785 nm laser. Spectra were collected with an exposure time of 60 s over 10 accumulations, covering the range of 400–2000 cm^−1^ at a resolution of 2 cm^−1^, and normalized against the phenylalanine band at 1003 cm^−1^ as an internal standard.

#### 2.4.2 The HNMR spectra

The nanoemulsion was diluted with D_2_O to achieve a final D_2_O concentration of 5% (*v*/*v*), followed by the addition of 0.1% (*w*/*v*) DSS as the chemical shift internal standard. Measurements were performed on a 500 MHz NMR spectrometer (Avance III 400 MHz, Bruker, Switzerland), and the resulting characteristic peak shifts and integration area changes were analyzed to probe interfacial molecular interactions.

### 2.5 The storage stability of nanoemulsion

A four-part analysis of the environmental stability of emulsions was conducted, including pH stability, temperature stability, enzyme stability and storage stability. The pH stability was evaluated with the adjustment of pH to 3.0–11.0 and kept at 25 °C for 24 h before testing. To investigate ionic stability, 2 mL of different concentrations of NaCl solutions were added to 3 mL of freshly prepared NaCl emulsions at concentrations of 0–500 mM. Before testing, the mixed emulsion was kept at 25 °C for 24 h. The enzyme stability was test with pepsin and trypsin, the enzyme concentration was adjusted with 1000 U/mL, and the emulsion was kept at 25 °C for 2 h. The storage stability was investigated by observing the freshly prepared emulsions at 40 °C for 60 days without adding NaCl solution. Finally, the psize of the emulsion was determined.

### 2.6 The in vitro simulated digestion

An in vitro gastrointestinal tract (GIT) model was established to simulate the oral-gastro-intestinal processing of the nanoemulsions. Simulated salivary fluid (SSF), simulated gastric fluid (SGF), and simulated intestinal fluid (SIF) were prepared according to previously reported methods. Briefly, the nanoemulsion was initially incubated with SSF (pH 6.8, containing KCl 15.10 mmol/L, KH₂PO₄ 3.70 mmol/L, NaHCO₃ 13.60 mmol/L, MgCl₂·6H₂O 0.15 mmol/L, (NH₄)₂CO₃ 0.06 mmol/L, CaCl₂·2H₂O 1.50 mmol/L, and α-amylase 1500 U/mL) at 37 °C for 10 min under constant agitation. Subsequently, an equal volume of SGF (pH 2.0, containing KCl 6.90 mmol/L, KH₂PO₄ 0.90 mmol/L, NaHCO₃ 25.00 mmol/L, (NH₄)₂CO₃ 47.20 mmol/L, MgCl₂·6H₂O 0.10 mmol/L, CaCl₂·2H₂O 0.15 mmol/L, and pepsin 25,000 U/mL) was added and incubated for 60 min to simulate gastric digestion. The intestinal digestion was then initiated by adding SIF (pH 7.0, containing KCl 6.80 mmol/L, KH₂PO₄ 0.80 mmol/L, NaHCO₃ 85.00 mmol/L, (NH₄)₂CO₃ 38.40 mmol/L, MgCl₂·6H₂O 0.33 mmol/L, CaCl₂·2H₂O 0.60 mmol/L, trypsin 800 U/mL, lipase 800 U/mL, and bile salts 0.5 wt%), and the pH was maintained at 7.0 using automated titration with 0.25 M NaOH for 2 h. The physicochemical properties of the emulsions, including droplet dimensions, surface charge, and microstructural morphology, were monitored at each digestive stage. Upon completion of intestinal digestion, the resulting digesta were subjected to ultracentrifugation at 40,000 ×*g* for 30 min to isolate the micellar phase. The concentration of salidroside entrapped within the micelles was determined by HPLC, and the bioaccessibility was expressed as the ratio of the compound recovered in the micellar fraction to the total compound present in the initial emulsion.

### 2.7 The bioavailability of salidroside

Caco-2 cells were seeded in 6-well plates at a density of 2 × 10^5^ cells/well and cultured for 48 h to allow attachment and partial differentiation. The culture medium was replaced with fresh serum-free DMEM containing micelles and incubated at 37 °C for 4 h. After incubation, the cells were rapidly washed three times with ice-cold PBS to terminate uptake and remove surface-bound salidroside. Cells were then detached with 0.25% trypsin-EDTA, collected by centrifugation at 1000 ×*g* for 5 min, and lysed in cell lysis buffer on ice for 30 min. The lysate was centrifuged at 12,000 ×*g* for 10 min at 4 °C, and the supernatant was collected. Salidroside content in the supernatant was quantified by HPLC. The bioavailability was calculated as amount of compound in cells to the total amount of compound in micelles.

### 2.8 Anti‑oxygen-induced damage in RAW264.7 cells by digestive micelles

RAW264.7 macrophages were routinely cultured in DMEM supplemented with 10% FBS and 1% penicillin-streptomycin at 37 °C in a 5% CO₂ incubator. Log-phase cells were adjusted to 5 × 10^4^ cells/mL and seeded into 96-well plates (100 μL/well) or at corresponding densities into 6-well plates and confocal dishes, followed by 24-h incubation to allow adherence. The experiment comprised two groups: control and micelle-treated. After 2-h pre-incubation with complete medium for the control group or the isolated micellar fraction from in vitro digestion, the control and micelle groups were exposed to 200 μM CoCl_2_ for 22 h to induce chemical hypoxia. Cell viability was determined using the CCK-8 assay by measuring absorbance at 450 nm with a microplate reader (SpectraMax iD3, Molecular Devices, San Jose, CA, USA). For live/dead staining, cells were washed with cold PBS, incubated with 2 μM Calcein-AM and 4 μM PI for 15 min at 37 °C in the dark, and imaged by CLSM. For the determination of MDA, SOD, ATP, HIF-1α, EPO and ET-1, a control group (No-added) was established, in which cells were cultured in complete medium alone, without the addition of micelles or CoCl₂, to serve as a control for normal physiological conditions. Intracellular ROS (reactive oxygen species) and NO (nitric oxide) levels were respectively detected using 10 μM DCFH-DA (20 min, Ex/Em = 488/525 nm) and 5 μM DAF-FM DA (20 min, Ex/Em = 495/515 nm), followed by CLSM analysis. For biochemical assays, cells were washed thrice with ice-cold PBS, lysed in pre-chilled lysis buffer on ice for 30 min, and centrifuged at 12,000 ×*g* at 4 °C for 10 min, the supernatants were collected for BCA protein quantification and subsequent ELISA determination of MDA, SOD, ATP, HIF-1α, EPO, and ET-1 according to the manufacturer's protocols.

### 2.9 Data analysis

With the exception of the rheological measurements, all experiments were repeated three times. For the rheological measurements, eight independently prepared nanoemulsion samples (*n* = 8) were used to enhance statistical reliability, taking full account of the high sensitivity of these measurements to sample preparation and loading conditions. The test data were analyzed using the LSD (Least Significant Difference) method in IBM SPSS software (version 21.0). Results are expressed as means with standard deviations (SDs), and multiple comparison tests were performed. A *p*-value of <0.05 was taken as indicating a statistically significant difference. Graphs of the test data were plotted using Origin Pro 2021 software.

## 3 Results and discussion

### 3.1 Mechanism of ultrasound-driven interfacial crosslinking

Fig. 8G illustrates the overall mechanism of action of this ultrasound-driven strategy. The ultrasound-induced cavitation effect promotes the refinement of nanoemulsion droplets and the uniform rearrangement of interfacial components, thereby increasing the oil–water interfacial area and providing more binding sites for the multiple interactions between chitosan and phosphate. Subsequently, chitosan and phosphate undergo synergistic assembly via ionic interactions, hydrogen bonding and coordination, forming a dense viscoelastic gel layer at the nanoemulsion interface, thereby constructing a stable core–shell structure and enhancing the interface barrier properties. This enhanced interface network not only improves the stable retention capacity of rhodiolin in the nanoemulsion system but also enables sustained release by regulating molecular diffusion processes. During gastrointestinal digestion, the stable cross-linked shell promotes the formation of mixed micelles loaded with rhodiolin, thereby improving its bioavailability; the subsequently released rhodiolin micelles can further exert antioxidant effects, laying the foundation for subsequent cell-protective effects.

### 3.2 Physicochemical properties of nanoemulsions

**Fig. 1:**
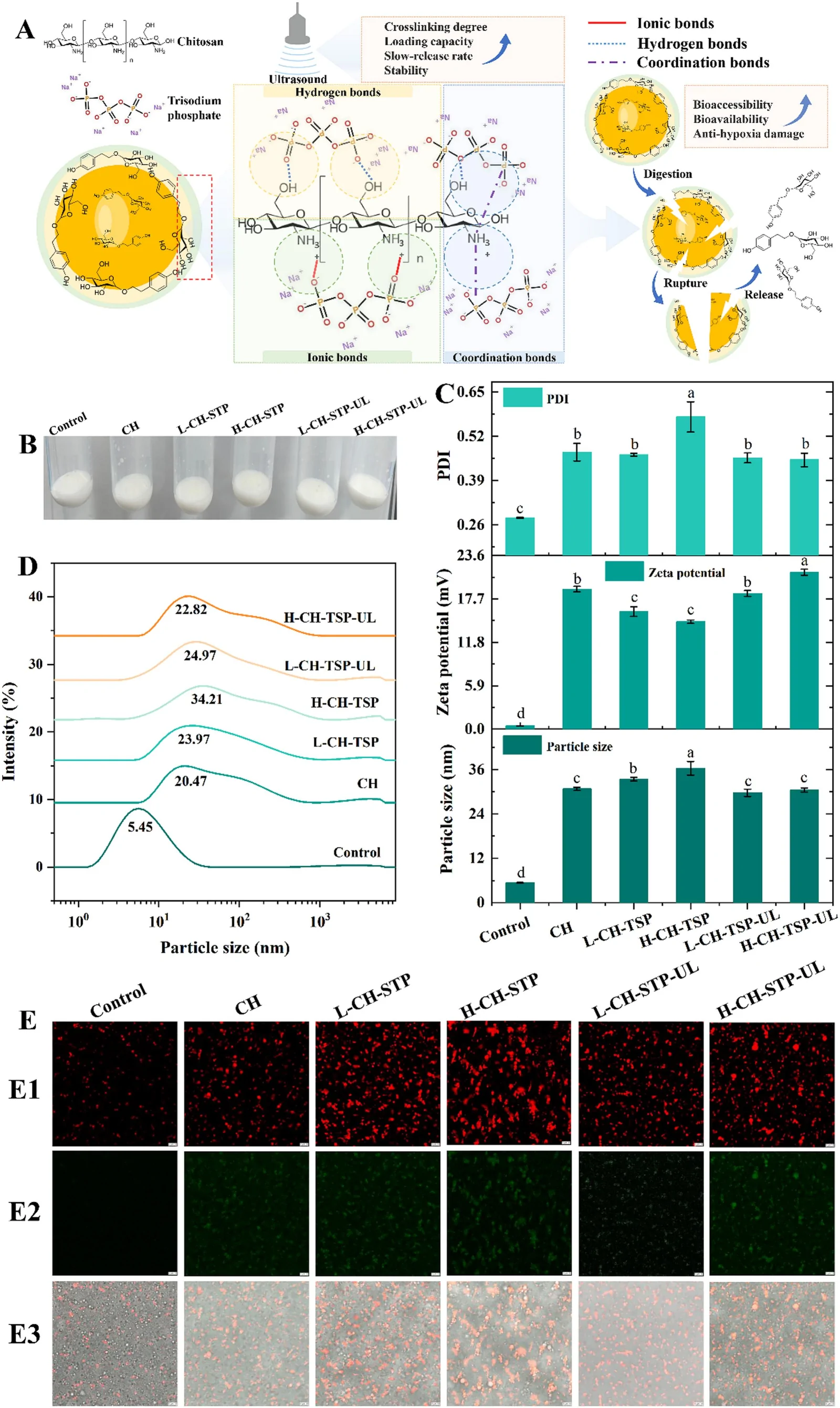
Sample preparation procedure and the proposed mechanism (A), Appearance features (B), size distribution (C), average droplet size, ζ-potential and PDI (D), and CLSM micrographs (E) of lecithin-stabilized nanoemulsions loaded with salidroside and treated with phosphate-crosslinked chitosan layers with or without ultrasound. (E1) Nile Red staining for lipids; (E2) FITC staining for chitosan; (E3) merged images with bright field. CH, chitosan-added emulsion; L-CH-TSP/H-CH-TSP, low/high-phosphate crosslinked chitosan-added nanoemulsion; L-CH-TSP-UL/H-CH-TSP-UL, low/high-phosphate crosslinked chitosan-added nanoemulsion subjected to ultrasound. Different lowercase letters indicate significant differences *(p* *<* *0.05)*. Same abbreviations apply to Fig. 2, Fig. 3, Fig. 4, Fig. 5, Fig. 6, Fig. 7, Fig. 8. (For interpretation of the references to colour in this figure legend, the reader is referred to the web version of this article.)

The appearance features, size distribution, psize, ζ-potential, PDI and CLSM microstructure are illustrated in Fig. 1. The control group exhibited a typical milky-white appearance with an average droplet size of 5.42 nm and a narrow distribution, indicating excellent monodispersity of the primary emulsion. Upon chitosan incorporation, the droplet size significantly increased to 30.81 nm, attributable to the thickened hydrated layer formed by chitosan adsorption at the droplet surface. Following the introduction of TSP for ionic crosslinking, the droplet size exhibited a dose-dependent increase, reaching 33.40 nm for L-CH-TSP and 36.39 nm for H-CH-TSP, reflecting the progressive construction of chitosan–phosphate crosslinked networks at the oil–water interface. The psize distribution peaks observed in chitosan-containing emulsion, Fig. 1B, further suggested enhanced inter-droplet bridging and increased contact sites between emulsion droplets. Notably, ultrasound significantly reduced droplet sizes (*p* < 0.05), although no significant difference was observed between L-CH-TSP-UL and H-CH-TSP-UL (*p* > 0.05). Ultrasound promoted rapid and uniform diffusion of phosphate ions within the interfacial layer, which homogenized crosslinking density rather than increasing shell thickness (Zhang et al., 2025).

ζ-potential and PDI reflected the surface charge characteristics and dispersion state of the droplets, as shown in Fig. 1C. The control group displayed a ζ-potential of 0.44 mV, indicating that the primary emulsion relied solely on steric stabilization with negligible electrostatic contribution, whereas chitosan incorporation significantly elevated the ζ-potential to 19.03 mV, confirming successful electrostatic anchoring of protonated amino groups at the droplet surface. The subsequent addition of TSP significantly decreased the ζ-potential, as phosphate ions partially neutralized the positive charges. This charge balance preserved sufficient electrostatic repulsion to prevent coalescence while providing the oppositely charged interactions required for ionic crosslinking. Ultrasound restored the ζ-potential to 18.43 mV and 21.33 mV for L-CH-TSP-UL and H-CH-TSP-UL, respectively, suggesting ultrasound-promoted redistribution and dense adsorption of chitosan at the interface with more amino groups exposed to the aqueous phase. All chitosan-containing samples exhibited significantly higher PDI values than the control (*p* < 0.05), originating from the molecular weight polydispersity of chitosan and the spatial heterogeneity of crosslinking reactions. Particularly, the H-CH-TSP group displayed the highest PDI, confirming microscale heterogeneity induced by high phosphate concentrations in the absence of ultrasound. In contrast, ultrasound reduced the PDI to 0.45, and combined with the highest ζ-potential and moderate droplet size, this indicated that ultrasound successfully optimized crosslinking network uniformity, yielding a highly charged and monodisperse stable system (Yue et al., 2022).

CLSM images of Nile Red-stained oil droplets are presented in Fig. 1D, the control group showed sparsely dispersed small oil droplets, whereas the CH group exhibited increased droplet density and slightly larger sizes corresponding to mild flocculation induced by weak chitosan adsorption. For L-CH-TSP and H-CH-TSP, oil droplets were distinctly enveloped by chitosan networks, with the H-CH-TSP group displaying obvious bridging flocculation where multiple droplets were trapped within the same chitosan network domain, directly correlating with the largest droplet size and highest PDI in this group and evidencing the formation of undesirable aggregates caused by excessive crosslinking. Ultrasound attenuated droplet bridging, and the CH group displayed diffuse background fluorescence (Fig. 1D2), indicating substantial free chitosan in the continuous phase, whereas TSP-crosslinked groups showed network-like aggregated fluorescence, demonstrating crosslinking-driven interfacial enrichment of chitosan from the bulk phase, and the ultrasound-treated groups exhibited more uniform interfacial network distributions(Li et al., 2025). Bright-field overlay images (Fig. 1D3) clearly illustrated the structural evolution from discrete oil droplets in the control, to bridged aggregates in H-CH-TSP, and finally to dispersed crosslinked networks in H-CH-TSP-UL.

### 3.3 Salidroside loading and release capacity

**Fig. 2:**
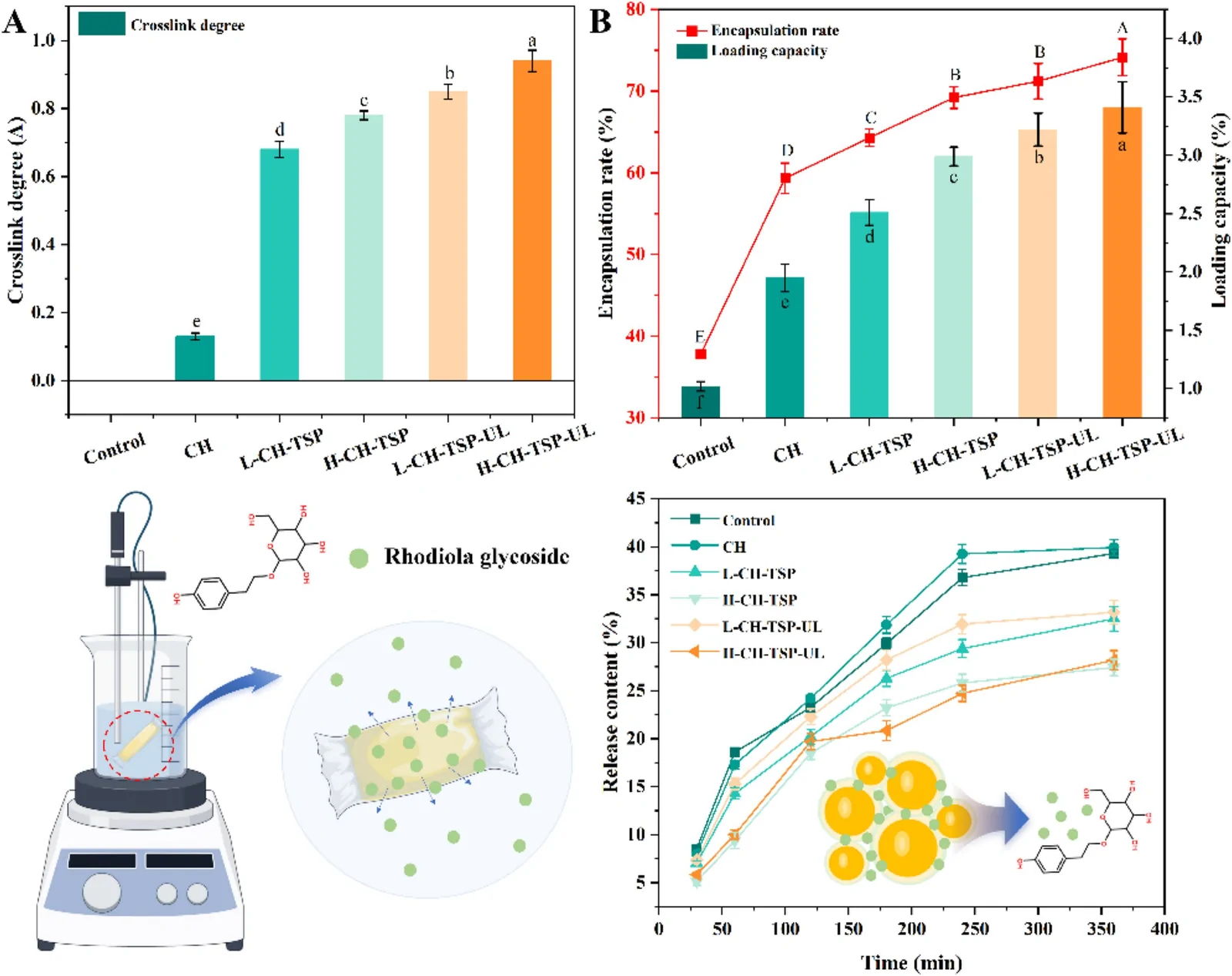
Crosslinking degree of chitosan (A), encapsulation efficiency and loading capacity (B), dialysis schematic (C), and salidroside release profiles (D) of nanoemulsions. Different lowercase letters indicate significant differences *(p* *<* *0.05).*

The crosslinking degree, loading capacity, and release content of salidroside in the nanoemulsions are shown in Fig. 2. The control group lacking chitosan was unable to form crosslinked networks, while the CH group exhibited only a low crosslinking degree attributable to weak physical crosslinking via inter-chain hydrogen bonding and entanglement rather than chemical ionic crosslinking (Wang et al., 2024). Upon TSP introduction, the crosslinking degree increased to 0.68 for L-CH-TSP and 0.78 for H-CH-TSP, indicating the formation of crosslinking nodes between multivalent phosphate ions and chitosan amino groups that constructed three-dimensional network structures. Ultrasound further significantly enhanced crosslinking degrees (*p* < 0.05), in the low-TSP nanoemulsion, phosphate diffusion was restricted and crosslinking suffered from kinetic heterogeneity, but cavitation-generated microturbulence and localized thermal fluctuations substantially accelerated phosphate mass transfer toward chitosan chains, activating more potential crosslinking sites (Faheem et al., 2026). In the high-TSP nanoemulsion, ultrasound primarily promoted uniform reorganization of the crosslinking network rather than simply increasing crosslinking density. The highest crosslinking degree achieved in the H-CH-TSP-UL group suggested that amino groups were almost fully crosslinked by phosphate ions under that condition, forming a highly dense and chemically stable interfacial gel network.

Loading capacity is presented in Fig. 2B, the control group exhibited the lowest encapsulation efficiency (EE) and loading capacity (DL), whereas chitosan incorporation increased EE and DL to approximately 59.34% and 1.95%, respectively, because the viscous interfacial layer created a kinetic barrier hindering salidroside diffusion from the aqueous to the oil phase, while hydroxyl and amino groups on chitosan chains formed weak hydrogen bonding interactions with the glycosidic hydroxyls of salidroside, prolonging salidroside residence time at the interface (Li et al., 2024). The L-CH-TSP group achieved EE and DL of 64.28% and 2.51%, respectively, whereas the H-CH-TSP group further improved these values to 69.21% and 2.99%, as the progressive densification of crosslinked networks significantly reduced interfacial permeability and minimized salidroside leakage. Simultaneously, the rigid crosslinked network effectively locked encapsulated salidroside, and the high crosslinking density may have generated adsorptive pores within the network. The ultrasound-treated group achieved the highest EE and DL, originating from the synergistic effect of ultrasound -induced droplet size reduction and interfacial densification, wherein smaller droplets provided greater specific surface area and oil–water interfacial area, while the uniform dense crosslinked shell maximized the interfacial barrier effect (Fig. 8G), enabling more efficient salidroside capture within the oil core and viscoelastic interfacial layer during emulsification (Cui et al., 2023).

The release kinetic curves are shown in Fig. 2C, with release patterns closely correlated with crosslinking degree and microstructure. The control and CH groups displayed typical burst-release profiles followed by rapid equilibration, with cumulative release exceeding 15% within 50 min and approaching 40% at 360 min, arising from loose interfacial structures that failed to effectively impede transmembrane diffusion of salidroside. The L-CH-TSP and H-CH-TSP groups exhibited significantly reduced release rates, with 360-min cumulative release decreasing to 32.49% and 27.42%, respectively, demonstrating clear sustained-release characteristics associated with increased diffusion pathway tortuosity and reduced interfacial permeability conferred by the crosslinked networks. It is worth noting that the effect of ultrasonic treatment on release behavior is closely related to the degree of cross-linking. No significant difference in final release rate was observed between L-CH-TSP-UL and its non-sonicated counterpart (*p* > 0.05), indicating that, at lower degrees of cross-linking, although ultrasonication promotes the reorganization of interfacial components and the homogenization of the cross-linked structure, it does not significantly alter the overall release level. The H-CH-TSP-UL group exhibited the lowest cumulative release, 25.18%, combined with the highest crosslinking degree, optimal microstructure, and highest salidroside loading in this group. This may be attributed to the fact that ultrasonication facilitated the uniform formation of a chitosan–triphosphate cross-linked network under high phosphate conditions, resulting in a denser and more stable interfacial barrier that further restricted the diffusion of salidroside. The chitosan-triphosphate cross-linked network is the primary factor determining the sustained-release behavior, whilst ultrasonication primarily serves to optimise the structure; by enhancing the uniformity and stability of the interfacial network, it further enhances the release control capabilities of the nanoemulsion system under suitable cross-linking conditions. The uniform and dense ionic cross-linked network provides sufficient physical barriers to retard salidroside diffusion while avoiding the structural heterogeneity caused by excessive crosslinking (Konovalova et al., 2023).

### 3.4 Rheological properties of nanoemulsions

**Fig. 3:**
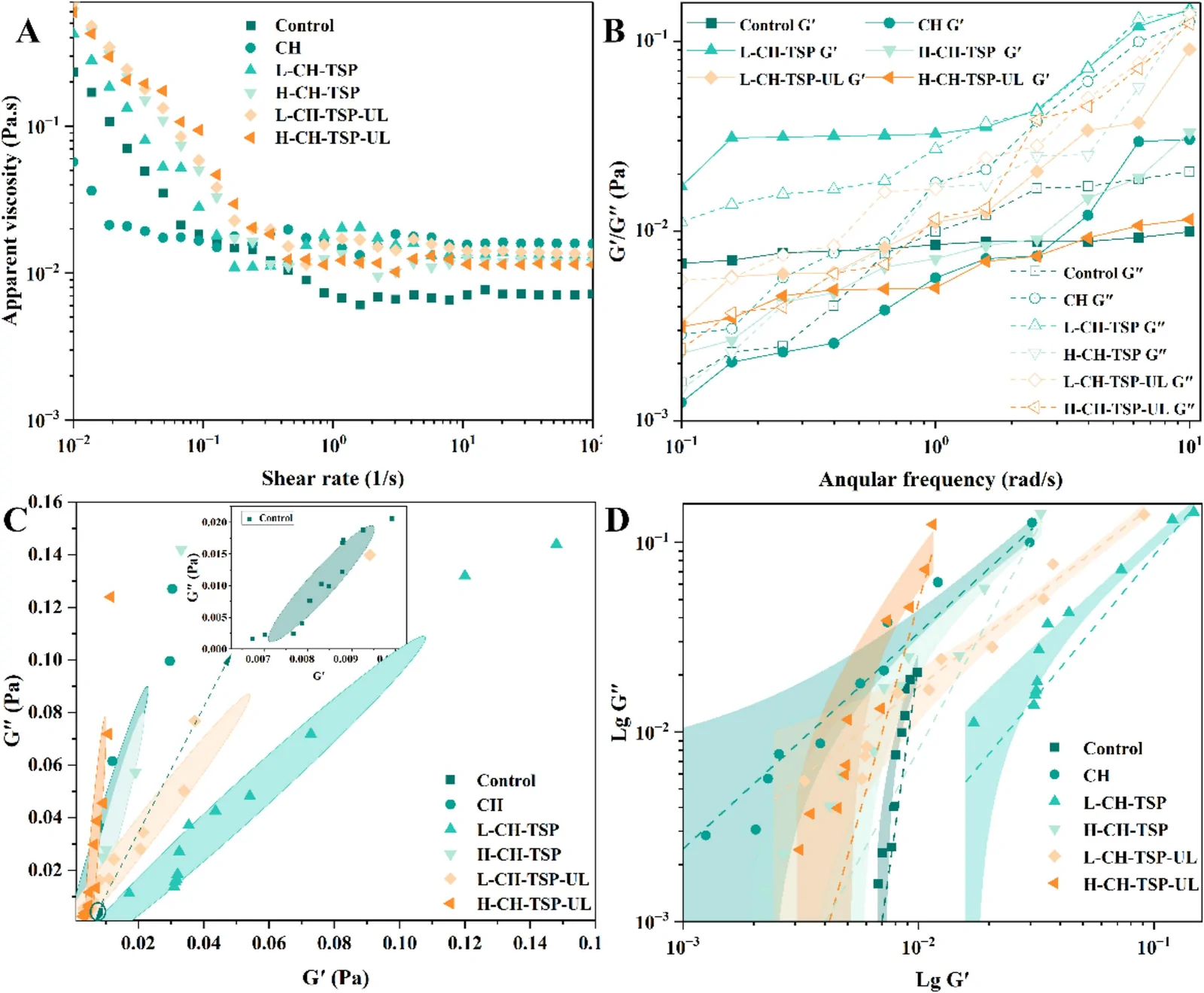
Apparent viscosity as a function of shear rate (A), storage modulus (G′) and loss modulus (G″) as a function of angular frequency (B), Cole–Cole plot (G″ vs. G′, C), and Han plot (log G″ vs. log G′, D) of nanoemulsions. Inset in (C): magnified view of the control group.

The rheological behavior of the nanoemulsions, as depicted in Fig. 3, revealed that all groups exhibited characteristic shear-thinning behavior, wherein apparent viscosity decreased with increasing shear rate, providing a favorable rheological basis for processing applications. The control group displayed the lowest viscosity with weak shear-thinning tendency. Chitosan incorporation significantly elevated viscosity, particularly in the low-shear region, attributable to physical entanglement and bridging of chitosan chains between oil droplets (Mourad et al., 2023). However, the CH group viscosity rapidly declined at high shear rates and approached that of the control, indicating facile disintegration of the physical entanglement network under shear with limited structural recovery. Both L-CH-TSP and H-CH-TSP groups maintained significantly higher viscosities than the CH group across the entire shear rate range, displaying more pronounced shear-thinning profiles. Notably, the H-CH-TSP group exhibited the highest viscosity in the low-shear region but converged with the L-CH-TSP group at high shear rates, suggesting that although its crosslinking network was dense, structural heterogeneity may have compromised its integrity under deformation. Following ultrasound, the L-CH-TSP-UL group displayed higher apparent viscosity than its non-sonicated counterpart in the low-shear region, whereas the H-CH-TSP-UL group showed no significant difference relative to H-CH-TSP.

Frequency sweep results are shown in Fig. 3B. Most groups exhibited G" dominant over G' throughout the frequency range, with both moduli increasing in parallel with frequency, characteristic of viscoelastic fluids dominated by viscous dissipation and lacking persistent elastic networks (Carvalho et al., 2022). The CH group showed lower G' and G" values than the control without significant narrowing of the inter-modulus gap, confirming that chitosan physical adsorption merely increased viscous resistance without establishing a true elastic skeleton. The L-CH-TSP group displayed a crossover between G' and G" in the mid-to-low frequency region, with G' trending toward a plateau at low frequencies, indicating the formation of a weak gel network. The H-CH-TSP group exhibited pronounced fluctuation and incomplete separation of G' and G" in the mid-to-high frequency region, associated with its higher PDI and bridging-flocculation microstructure, whereby heterogeneous psize distribution and network architecture led to superimposed multiscale relaxation processes. In contrast, the H-CH-TSP-UL group showed lower frequency dependence of G' compared with L-CH-TSP-UL, accompanied by reduced modulus values, suggesting that ultrasound induced network reorganization in the high-phosphate system with significantly diminished topological defects, yielding a more uniform elastic architecture.

Cole-Cole plots (Fig. 3C), mapping G" against G' in the complex plane, provided fingerprint information regarding the relaxation time distributions. An ideal Maxwell fluid should exhibit a semicircular profile, whereas systems with polydisperse relaxation times deviate from this geometry (Rolere et al., 2017). The control group displayed a flattened ellipse approximating a semicircle, indicating relatively simple relaxation mechanisms dominated by weak inter-droplet interactions. The CH group showed slight deviation from the semicircle but retained substantial curvature in the low-G' region, confirming that physical adsorption introduced minor long-range relaxation modes while the system remained predominantly viscous. For L-CH-TSP and H-CH-TSP, the Cole-Cole plots exhibited tailing or asymmetric extension, particularly with significantly elevated values in the high-G' region relative to the ideal semicircle, evidencing that the chitosan–phosphate crosslinked networks introduced a broad relaxation time spectrum spanning from molecular segmental motions to entire network rearrangements. The H-CH-TSP plot showed the greatest deviation with irregular fluctuations, corroborating its structural heterogeneity. The H-CH-TSP-UL plot was notably narrower, demonstrating that ultrasound narrowed the relaxation time distribution and homogenized the network structure through cavitation-mediated interfacial reorganization.

Han plots (Fig. 3D), presenting the linear relationship between G" and G' on double-logarithmic coordinates, revealed the viscoelastic transitions of the nanoemulsion systems (Porkodi et al., 2020). For homogeneous viscoelastic fluids, Han plots typically exhibit a slope approaching unity, whereas structured systems with developed elastic networks deviate from this linearity. The CH group displayed a lower slope than the control, reflecting incipient network formation, while the H-CH-TSP group showed altered slope behavior with increased scatter, indicating complex multiscale relaxation associated with crosslinked network development. The ultrasound-treated H-CH-TSP-UL group exhibited the most linear distribution with minimal scatter among all chitosan-containing formulations, accompanied by slope characteristics consistent with a more homogeneous viscoelastic structure. Collectively, these data demonstrate that chitosan–phosphate ionic crosslinking at the oil–water interface constructed a three-dimensional viscoelastic network, transforming the nanoemulsions from viscous fluids into viscoelastic weak gels, with ultrasound optimization ultimately yielding a uniform weak gel network architecture (Yue et al., 2022).

### 3.5 Molecular interactions in nanoemulsions

**Fig. 4:**
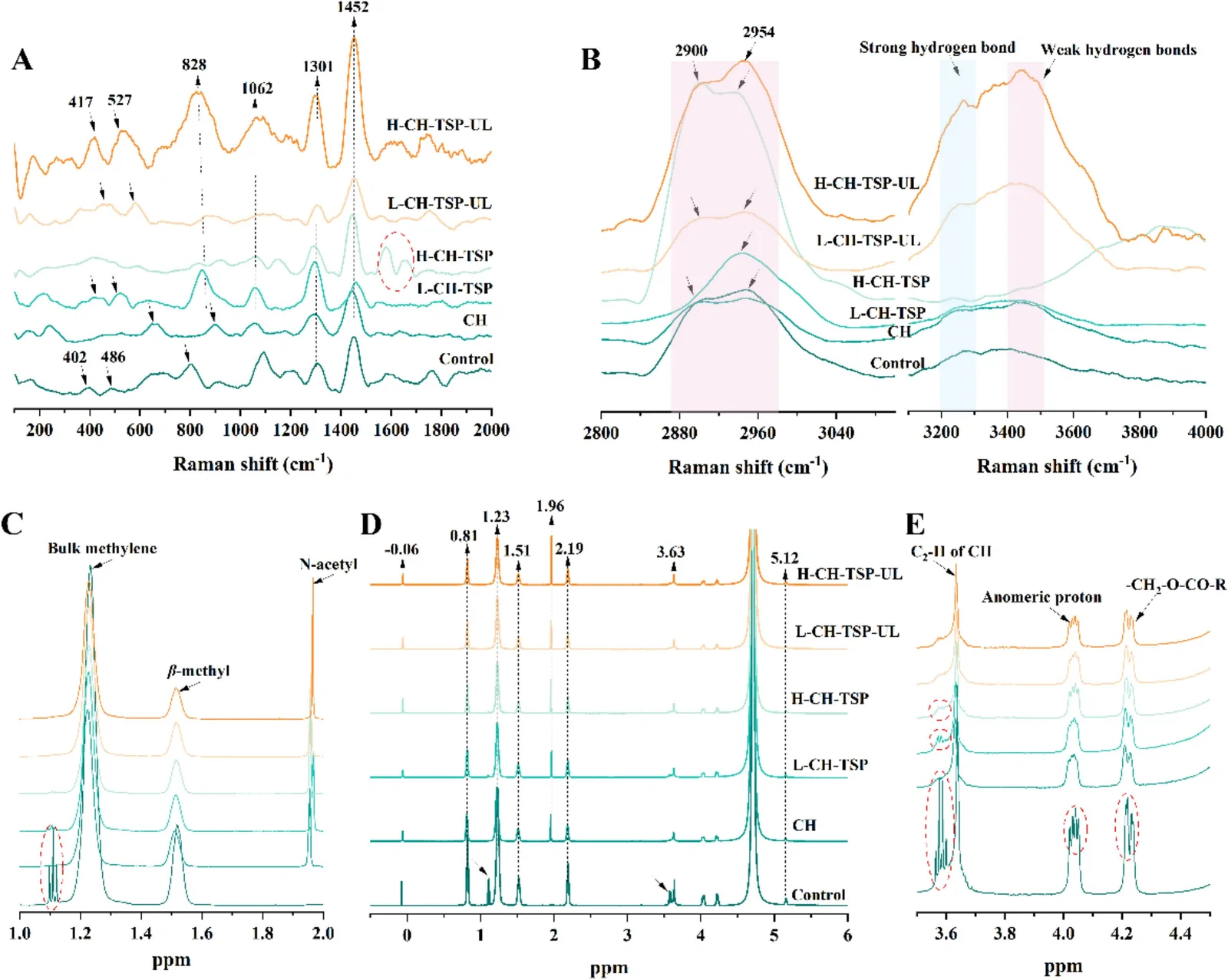
Raman spectra in the fingerprint region (200–2000 cm^−1^, A) and high-wavenumber region (2800–4000 cm^−1^, B), and ^1^H NMR spectra (C–E) of nanoemulsions. (C) Full spectrum (−0.5–6 ppm); (D) MCT oil fingerprint region (1.0–2.0 ppm); (E) anomeric proton region (3.5–4.5 ppm).

Raman spectroscopy was shown in Fig. 4A-B. In the fingerprint region (200–2000 cm^−1^, Fig. 4A), the CH₂ bending vibration of medium-chain triglyceride (MCT) oil at 1452 cm^−1^ appeared markedly enhanced with peak sharpening in the H-CH-TSP-UL group, indicating that the tightly wrapped chitosan–phosphate shell restricted random thermal motions of acyl chains and promoted more ordered packing conformations at the interface (Martins et al., 2023). More significantly, the band at approximately 1062 cm^−1^, assignable to the overlapping signals of chitosan C–O–C glycosidic linkage stretching and phosphate ν_3_ asymmetric stretching, exhibited a blue shift with increased half-bandwidth in TSP-crosslinked groups, confirming that phosphate ions established coupled hydrogen bonding and ionic interactions with chitosan hydroxyl and amino groups, thereby increasing the force constant of the glycosidic bonds. In the high-wavenumber region (2800–4000 cm^−1^, Fig. 4B), The O–H/N–H stretching vibration region provided information on hydrogen-bonding networks, the control and CH groups displayed narrow weak hydrogen-bonding peaks in the 3200–3600 cm^−1^ range. Whereas TSP-crosslinked groups, particularly H-CH-TSP-UL, showed significant red-shifting with enhanced intensity in this region, accompanied by synchronous intensification of the C—H stretching vibration near 2900 cm^−1^ and the 1452 cm^−1^ band, demonstrating that the crosslinked networks established extensive hydrophilic hydrogen-bonding networks associated with high crosslinking density and viscoelastic performance (Mahaninia & Wilson, 2017).

^1^H NMR spectroscopy (Fig. 4C–E) was employed to resolve the restricted dynamics of oil-core molecules and the interfacial chemical state of chitosan. The 1.0–2.0 ppm region constitutes the fingerprint region of MCT oil, the control group exhibited a characteristic triplet at 1.1 ppm, which was severely attenuated in chitosan-containing and ultrasound-treated nanoemulsions. When the chitosan–phosphate shell tightly encapsulates oil droplets, the isotropic rapid rotation of MCT molecules in the interfacial and near-interfacial regions is severely hindered. At approximately 1.96 ppm, chitosan-containing groups displayed a characteristic peak attributable to the *N*-acetyl group (–COCH_3_) of chitosan, whose intensity correlated positively with chitosan concentration, whereas the control showed negligible signal at this position. In the anomeric proton region of 3.6–4.4 ppm (Fig. 4E), the C_2_—H peak at 3.6 ppm shifted downfield following TSP addition, indicating protonation of –NH_2_ to –NH_3_^+^ and ion-pair formation with phosphate ions, which reduced the electron cloud density around the C_2_—H nucleus (Abouricha et al., 2025). Meanwhile, the chemical shift of MCT sn-1,3 glycerol backbone protons at approximately 4.2 ppm remained stable across all groups, combined with the observation in Fig. 4C that MCT fingerprint peaks exhibited only relaxation broadening without chemical shift changes, this collectively demonstrates that the chitosan network anchored primarily at the oil–water interface without engaging in significant intermolecular interactions with the oil-phase core. Full-spectrum results (Fig. 4D) showed that the control group possessed significantly stronger peaks at −0.06, 1.23, 1.51, 2.19, and 3.63 ppm compared with other groups. This relative intensity attenuation in crosslinked formulations originated from the relaxation shielding effect induced by the chitosan–phosphate interfacial layer, whereby the crosslinked network restricted the motional freedom of interfacial MCT molecules, leading to shortened relaxation times and reduced peak heights (Wang & Liu, 2014). In summary, chitosan and phosphate constructed a dense viscoelastic interfacial layer on droplet surfaces through ionic bonding, hydrogen-bonding networks and coordination bonds (Fig. 8G). The interfacial layer reduced oil-phase molecular mobility through physical confinement, forming a core–shell architecture characterized by high crosslinking density, broad linear viscoelastic regions, and enhanced structural uniformity, with ultrasound treatment further optimizing the homogeneity and density of the crosslinked network.

### 3.6 Environmental stress stability of nanoemulsions

**Fig. 5:**
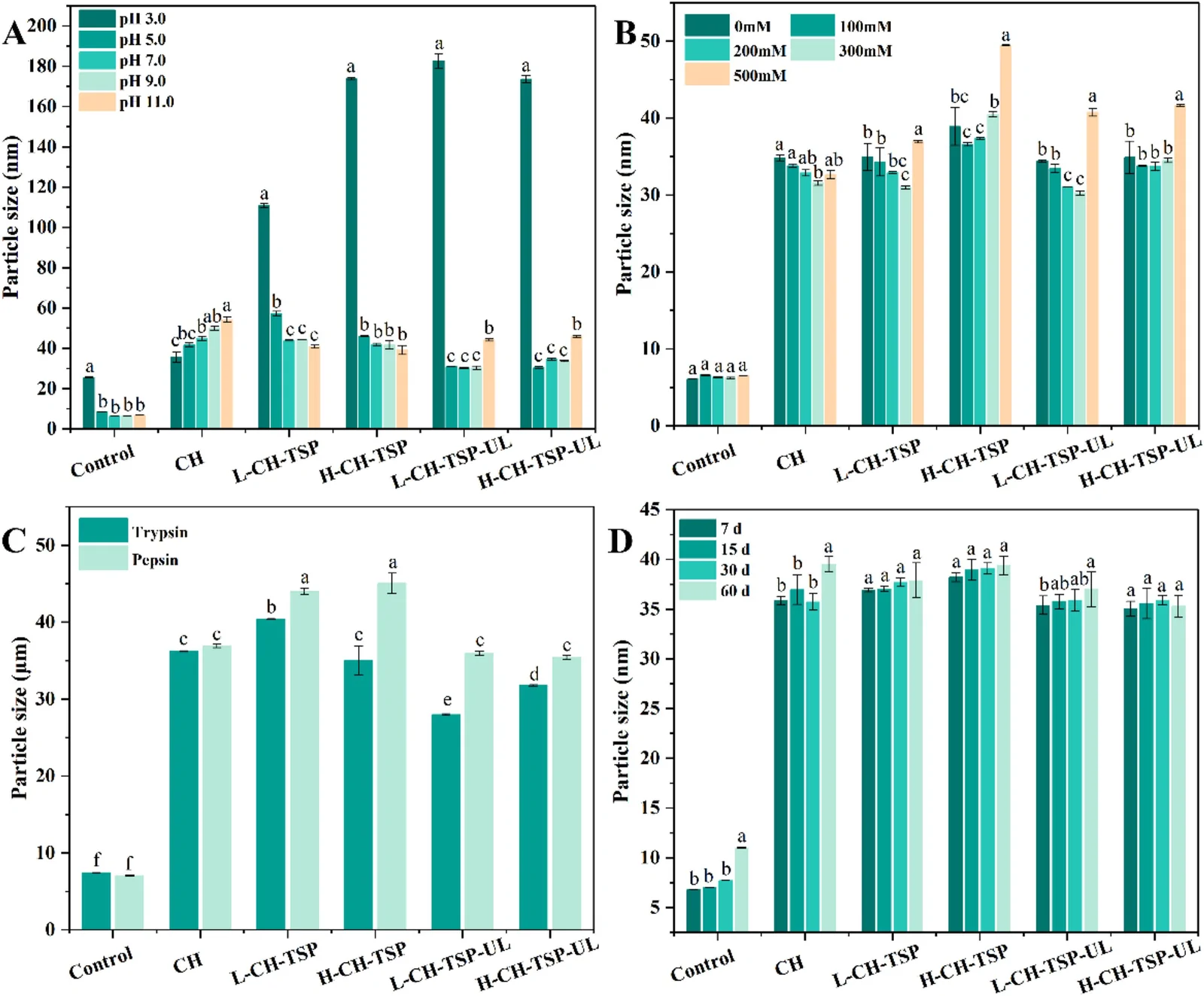
Environmental stability of nanoemulsions: pH-dependent stability (3.0–11.0, A), ionic strength stability (0–500 mM NaCl, B), enzymatic stability against pepsin and trypsin (C), and storage stability at 40 °C over 60 days (D). Different lowercase letters indicate significant differences *(p* *<* *0.05).*

The environmental stability of nanoemulsions dictates the applicability and biological functionality. pH-dependent stability (Fig. 5A) revealed that the CH group exhibited the smallest droplet size at pH 3.0, with size increasing monotonically as pH rose (*p* < 0.05), because complete protonation of chitosan amino groups under acidic conditions maximized charge density and electrostatic repulsion, whereas progressive deprotonation under alkaline conditions eliminated electrostatic stabilization, leaving only steric hindrance and causing droplet swelling. Conversely, TSP-crosslinked groups displayed an inverse trend, both L-CH-TSP and H-CH-TSP showed drastic size enlargement at pH 3.0 (*p* < 0.05), significantly exceeding the dimensions under neutral conditions, because excessive protonation of –NH_3_^+^ generated strong intramolecular electrostatic repulsion that disrupted network compactness, while excess H^+^ competed with phosphate ions for binding sites, weakening ionic crosslinks and triggering shell swelling (Liu et al., 2012). Under neutral to weakly alkaline conditions, all TSP-crosslinked formulations maintained stable droplet sizes (*p* > 0.05), as partial deprotonation of chitosan coupled with multivalent negative charges of phosphate ions established an electrostatic–ionic synergistic stabilization that preserved sufficient electrostatic repulsion while maintaining network rigidity. Under strongly alkaline conditions, all sonicated nanoemulsions exhibited significant size increases (*P* < 0.05), because near-complete deprotonation abolished electrostatic repulsion, leaving only steric hindrance and hydrophobic interactions, thereby compromising the charge-dependent stability of the crosslinked network.

Ionic strength stability is shown in Fig. 5B, the control group maintained constant droplet size across 0–500 mM NaCl, as its charge-neutral nature rendered it insensitive to ionic strength. The CH, L-CH-TSP, and L-CH-TSP-UL groups exhibited monotonically decreasing sizes within 0–300 mM NaCl (*p* < 0.05), with the L-CH-TSP and L-CH-TSP-UL groups showing significant size increases at 500 mM NaCl (*p* < 0.05), whereas the H-CH-TSP group displayed significant enlargement across 100–500 mM NaCl (*p* < 0.05). In contrast, the H-CH-TSP-UL group showed no significant size variation at 100–300 mM NaCl (*p* > 0.05), because high concentrations of Na^+^ screened the positive charges of –NH_3_^+^, weakening electrostatic repulsion, while ionic crosslinks underwent dynamic dissociation under elevated ionic strength (Li et al., 2020). The dense, highly crosslinked network optimized by ultrasound maintained structural integrity through physical entanglement and steric hindrance even when electrostatic repulsion was screened.

Enzymatic stability is presented in Fig. 5C, under pepsin treatment, all groups except H-CH-TSP and L-CH-TSP-UL exhibited significant size increases (*p* < 0.05), with the latter two groups showing size reduction relative to their original droplet dimensions. Under trypsin treatment, all groups displayed significant size enlargement (*p* < 0.05). Comparing pepsin versus trypsin treatments, no significant difference was observed between the control and CH groups (*p* > 0.05), whereas all other groups showed significantly larger droplet sizes after trypsin treatment than after pepsin treatment (*p* < 0.05). Pepsin degradation of chitosan proceeded primarily through nonspecific hydrolysis of glycosidic bonds and attack on uncrosslinked amino groups(Niu et al., 2019). The reduction in psize induced by trypsin may be attributed to the restructuring of the interfacial layer and the reorganization of the chitosan-triphosphate network structure. During digestion, the redistribution of interfacial components and the reduction in the degree of chitosan protonation may alter the charge distribution and network conformation of the interfacial layer, thereby modulating inter-droplet interactions and influencing changes in psize(McClements & Li, 2010). At the same time, the outer chitosan chains with weaker bonds within the cross-linked network may undergo dissociation or rearrangement preferentially, whilst the stable internal cross-linked structure is retained, thereby maintaining the structural integrity of the droplets.

Storage stability is shown in Fig. 5D, the control group showed no significant size change during the first 30 days (*p* > 0.05), but increased to 11.06 nm by day 60, the CH group followed a trend similar to the control. The L-CH-TSP, H-CH-TSP, and H-CH-TSP-UL groups exhibited insignificant size variations throughout storage (*p* > 0.05), indicating that dense crosslinked networks effectively inhibited Ostwald ripening. In contrast, the L-CH-TSP-UL group showed a significant difference between day 1 and day 60 (*p* < 0.05), reflecting that although low-concentration crosslinking combined with ultrasound improved short-term dispersibility, insufficient crosslinking density led to gradual network relaxation during long-term storage.

### 3.7 In vitro simulated gastrointestinal digestion

**Fig. 6:**
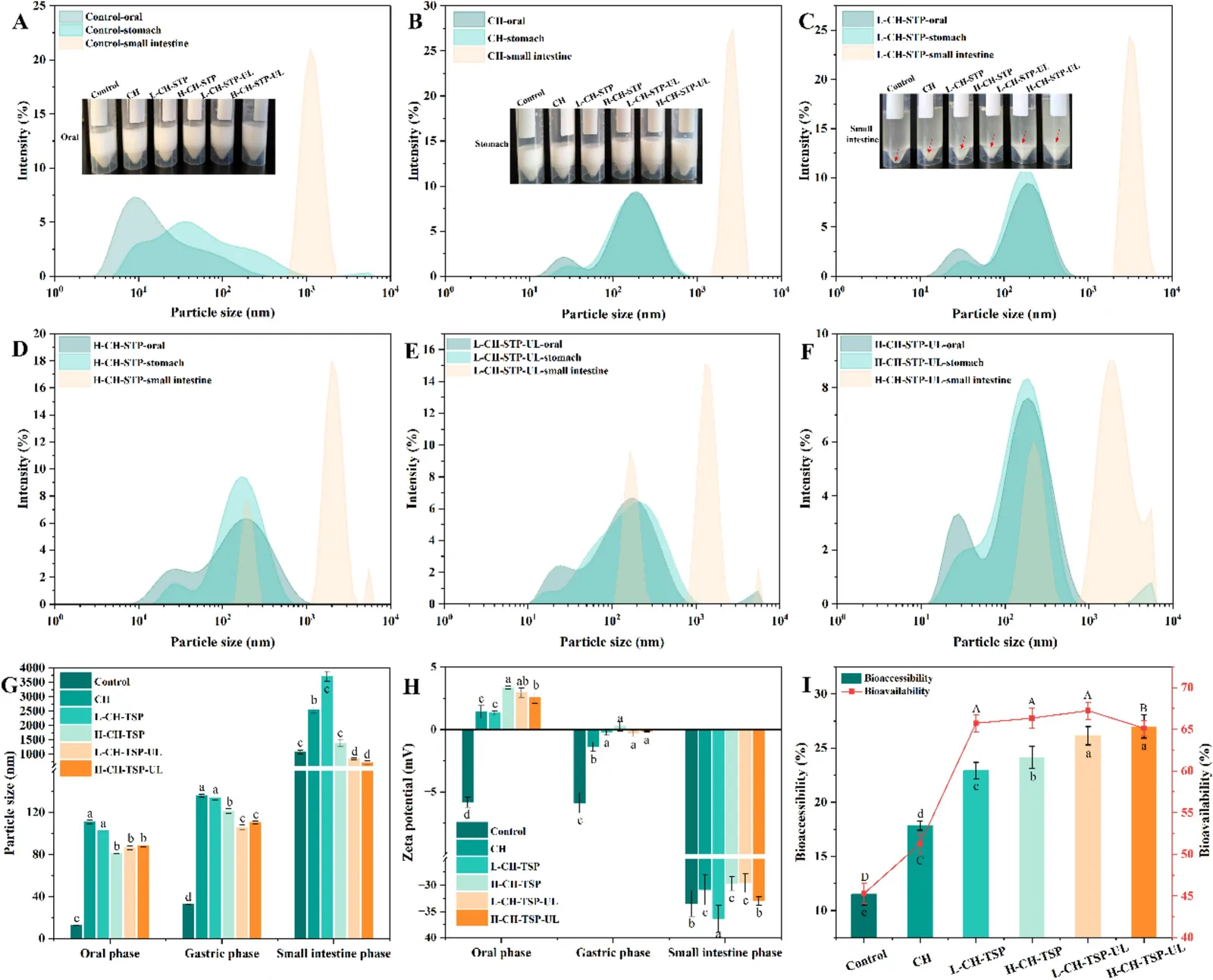
In vitro simulated gastrointestinal digestion of nanoemulsions. Droplet size distribution during oral, gastric, and intestinal phases (A–F), average droplet size (G), and ζ-potential (H). (I) Salidroside bioaccessibility after digestion and bioavailability across Caco-2 cell monolayers. Different lowercase letters indicate significant differences *(p* *<* *0.05).*

The dynamic evolution of droplet size during in vitro simulated digestion is illustrated in Fig. 6A–G. During the oral phase, all groups maintained bimodal size distributions, except for the control group, the other groups showed patterns generally consistent with those observed in the gastric phase but with more concentrated peak distributions, consistent with the expected limited impact of salivary amylase on MCT oil-based emulsions (Wang et al., 2023). Upon entering the gastric phase, all groups exhibited significant size increases (*p* < 0.05), the CH and L-CH-TSP groups displayed the largest sizes, because complete protonation of chitosan under strongly acidic conditions eliminated electrostatic repulsion, while pepsin hydrolysis of uncrosslinked chitosan chains compromised interfacial integrity. No significant difference was observed between the L-CH-TSP-UL and H-CH-TSP-UL groups (*p* > 0.05), and both were more stable than the other chitosan-containing groups, demonstrating that the dense crosslinked network optimized by ultrasound exhibited restricted swelling behavior in the gastric environment, wherein high crosslinking density preserved mechanical integrity and prevented catastrophic structural collaps. During the intestinal phase, the control, CH, and L-CH-TSP groups displayed unimodal size distributions, whereas all other groups exhibited bimodal or multimodal distributions. Among these, the L-CH-TSP group showed the largest average size, while the L-CH-TSP-UL and H-CH-TSP-UL groups displayed the smallest average sizes. Visible phase separation was observed in groups with insufficient interfacial protection, because oil droplets lacking adequate crosslinked shells underwent severe lipolysis and aggregation under the action of bile salts and pancreatic lipase. The crosslinked network in the intestinal environment underwent sacrificial degradation, with outer chitosan layers preferentially degraded by pancreatic enzymes, exposing positively charged inner cores that interacted with bile salts to form mixed micelles (Hu et al., 2021).

The ζ-potential evolution during digestion is shown in Fig. 6H. During the oral phase, except for the control group which maintained negative surface charge, all chitosan-containing groups exhibited significant positive potentials, confirming successful coating of droplet surfaces by positively charged chitosan–phosphate complexes and the formation of a stable cationic interfacial layer. Upon entering the gastric phase, chitosan-containing emulsions exhibited ζ-potentials near 0 mV, whereas the control group remained negatively charged at −5.87 mV. No significant difference was detected among the TSP-crosslinked groups (*p* > 0.05). Extensive protonation of amino groups under strongly acidic conditions caused polymer chain extension via intramolecular charge repulsion. However, the absence of high potentials indicated charge screening effects. Notably, the low-potential state of crosslinked groups in gastric fluid did not induce severe flocculation, implying that the dense ultrasound-optimized crosslinked network stabilized droplets primarily through steric hindrance rather than electrostatic repulsion alone (Wang & Heuzey, 2016). Upon entering the intestinal phase, all groups shifted to negative ζ-potentials with significant decreases (*p* < 0.05). Bile salts, as strongly anionic surfactants, adsorbed onto droplet interfaces through hydrophobic interactions, displacing portions of the original cationic chitosan layer and dominating surface charge characteristics. Concurrently, pancreatic lipase hydrolysis disrupted the emulsion interface, releasing free fatty acids that ionized in the intestinal environment. As outer chitosan layers were enzymatically degraded or displaced, internal structures were exposed and interacted with bile salts to form mixed micelles.

### 3.8 The bioaccessibility and bioavailability of salidroside

The bioaccessibility and bioavailability are presented in Fig. 6I. The control group exhibited the lowest bioaccessibility and bioavailability, attributable to the absence of interfacial protection, whereby MCT oil droplets suffered bile salt displacement and aggressive lipase attack during the gastric phase, causing extensive salidroside leakage and enzymatic deactivation prior to reaching absorption sites. Although the CH group provided certain steric hindrance through the chitosan physical adsorption layer, delaying lipid hydrolysis, its barrier effect remained relatively loose with limited improvement in salidroside protection efficiency (Shah et al., 2016). In contrast, the L-CH-TSP and H-CH-TSP demonstrated significantly enhanced bioaccessibility (*p* < 0.05), and the ultrasound-optimized L-CH-TSP-UL and H-CH-TSP-UL groups achieved optimal bioaccessibility without significant difference between them (*p* > 0.05), although both outperformed the non-ultrasonic counterparts. Caco-2 cells were employed to evaluate the bioavailability of salidroside in micelles, revealing the lowest rate in the control group, followed by the CH group, with no significant difference among L-CH-TSP, H-CH-TSP, and L-CH-TSP-UL (*P* > 0.05), whereas H-CH-TSP-UL was slightly lower. During the gastric phase, the highly crosslinked chitosan–phosphate network formed a dense physical barrier that effectively inhibited pepsin erosion and salidroside burst release. Upon entering the intestinal phase, the crosslinked network underwent controlled swelling and degradation under pancreatic enzyme and alkaline conditions, permitting pancreatic lipase access to the oil core for hydrolysis while stabilizing mixed micelles through electrostatic interactions. This prevented excessive micellar aggregation or precipitation and significantly enhanced salidroside solubility in the micellar phase. Ultrasound endowed the emulsion with more uniform droplet size distribution and denser crosslinked structure, eliminating localized premature rupture caused by structural defects in non-ultrasonic groups. More importantly, positively charged chitosan degradation fragments or complex micelles could bind tightly to negatively charged Caco-2 cell membranes via electrostatic attraction, prolonging salidroside residence time at the absorption interface and potentially enhancing transmembrane transport efficiency through tight junction opening or promoted endocytosis (Vors et al., 2012). However, the excessively dense and rigid network formed by over-crosslinking, while providing physical barrier protection, hindered salidroside release in the intestinal environment, manifested as the decreased transmembrane transport in H-CH-TSP-UL.

### 3.9 The improvement effect of salidroside in micelles on the anaerobic damage of RAW264.7 cells

**Fig. 7:**
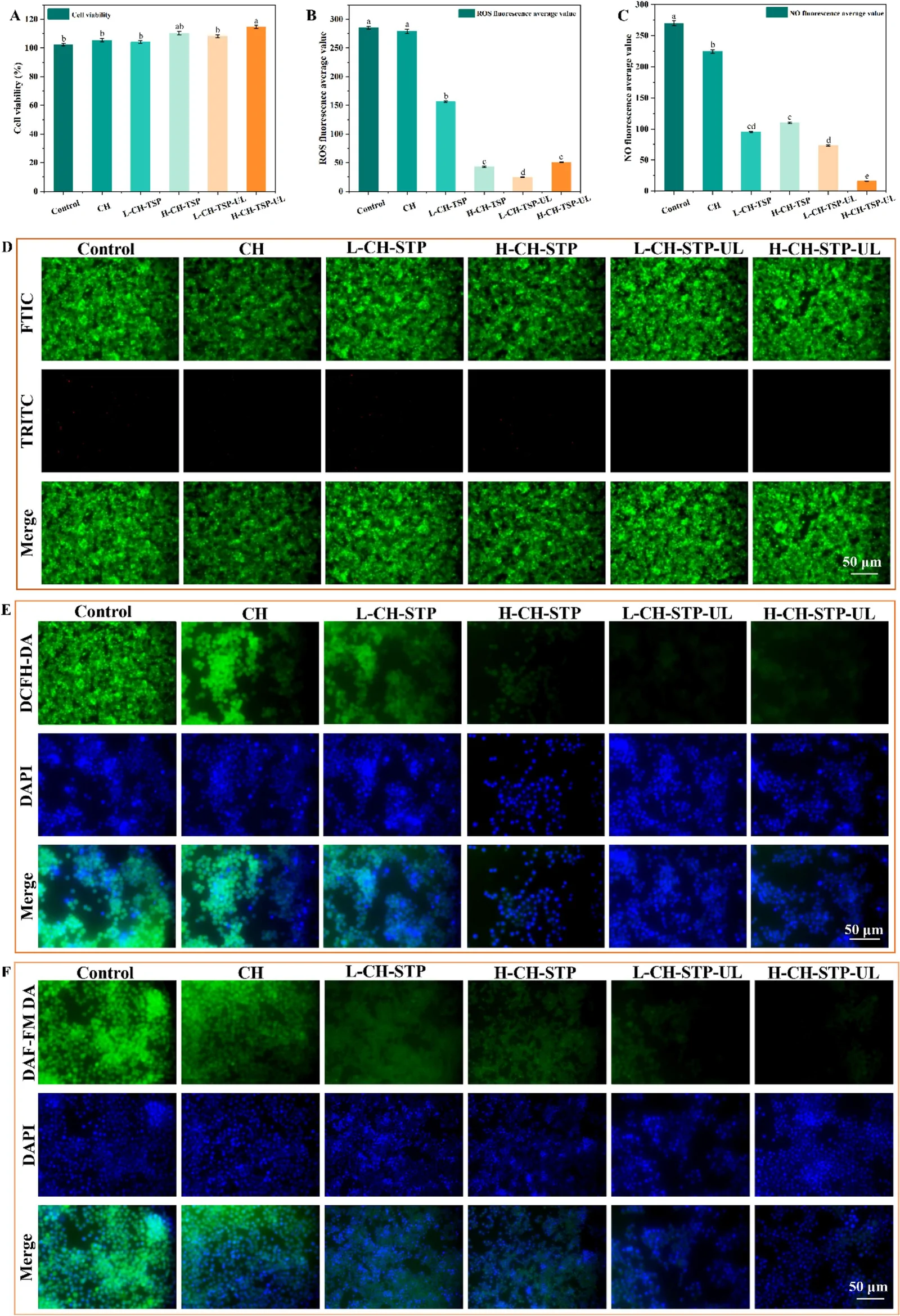
Cytoprotective effects of digested micelles on RAW264.7 macrophages under hypoxic conditions. Cell viability (A), intracellular ROS (B), and NO levels (C). Representative CLSM images: live/dead staining (Calcein-AM/PI), intracellular ROS (DCFH-DA), NO (DAF-FM DA), and nuclear staining (DAPI).

Cell viability (Fig. 7A) and live/dead fluorescence staining (Fig. 7D) results showed that all salidroside-containing groups maintained extremely high cellular viability (>95%), and no TRITC-labeled death signals were detected in the L-CH-TSP-UL and H-CH-TSP-UL groups, confirming the excellent biocompatibility of post-digestion micelles. Nevertheless, significant differences were observed among formulations regarding oxidative stress modulation. ROS (Fig. 7B, E) and NO (Fig. 7C, F) detection results indicated that the control and CH groups, lacking effective interfacial crosslinking, likely experienced salidroside burst release during early digestion, leading to partial salidroside degradation or dilution before reaching cells, and consequently maintained high intracellular ROS and NO levels. Conversely, the TSP-crosslinked groups, through the dense network formed by phosphate and chitosan, conferred sustained-release and protective effects during digestion. The crosslinked layer effectively prevented premature salidroside leakage and oxidative deactivation in simulated digestive fluids, with the L-CH-TSP-UL group showing the lowest ROS fluorescence, while no significant difference was observed between the H-CH-TSP and H-CH-TSP-UL groups (*p* > 0.05). Ultrasound further optimized droplet size uniformity and interfacial compactness, enabling the formed mixed micelles to possess superior colloidal stability. This stable micellar carrier continuously and efficiently delivered salidroside to the cell periphery, maintaining effective intracellular salidroside concentrations, thereby significantly suppressing hypoxia-induced ROS burst and NO overproduction (*p* < 0.05) and blocking the polarization pathway of macrophages toward the pro-inflammatory M1 phenotype (Guo et al., 2023). These results further demonstrate that the degree of interfacial cross-linking and ultrasonic modulation do not merely increase the drug loading capacity, but rather enhance the utilization efficiency of rhodiolin at the cellular level by optimizing structural stability and the release behavior of the active ingredient during the digestive process. Furthermore, the lower levels of ROS and NO indicate that the ultrasonically optimized core–shell nanoemulsion system is more effective at protecting rhodiolin from degradation in the digestive environment and promoting its antioxidant stress-relieving effects.

**Fig. 8:**
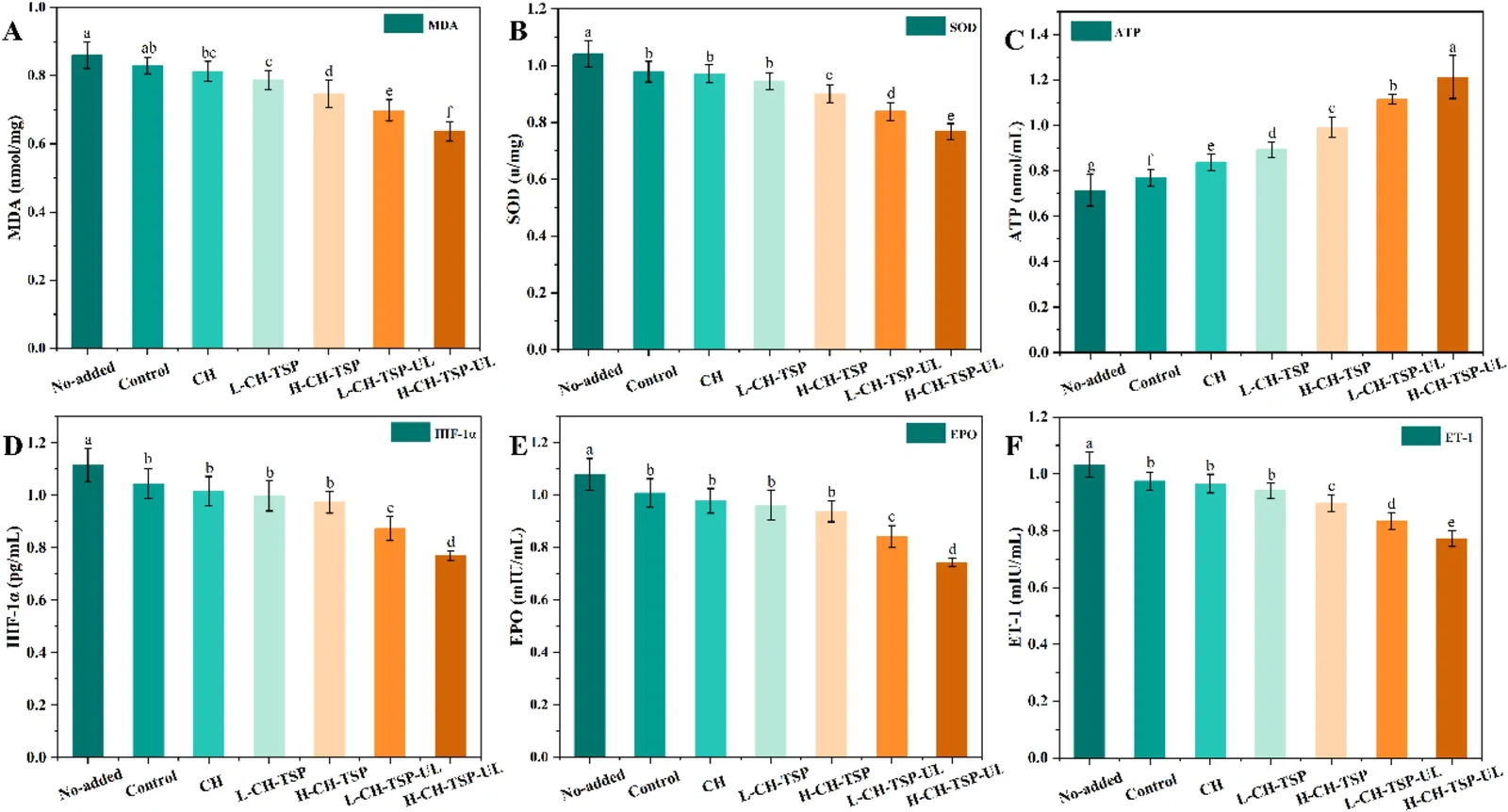
Biochemical and molecular markers in RAW264.7 cells after treatment with digested micelles. MDA (A), SOD (B), ATP (C), HIF-1α (D), EPO (E), and ET-1 (F) levels.

Fig. 8 further revealed the regulatory effects of different nanoemulsion structures on cellular oxidative damage and energy metabolism. The gradient reduction in malondialdehyde (MDA, Fig. 8A) levels, particularly reaching the minimum in the H-CH-TSP-UL group, confirmed that the dense interfacial layer formed by high crosslinking density combined with ultrasound homogenization maximally protected salidroside through the digestive environment and effectively scavenged intracellular free radicals, thereby interrupting the chain reaction of lipid peroxidation. Regarding superoxide dismutase (SOD, Fig. 8B) activity, the elevated level in the No-added group reflected compensatory upregulation in response to hypoxic stress, whereas the significantly reduced SOD activity in the H-CH-TSP-UL group did not indicate weakened antioxidant capacity. It demonstrated that salidroside directly scavenged ROS, alleviated cellular stress, and eliminated the need to maintain energy-consuming SOD overexpression, thereby restoring normal metabolic homeostasis. ATP levels (Fig. 8C) showed that the H-CH-TSP-UL group was significantly higher than other groups, indicating that this delivery system not only reduced oxidative damage but also protected mitochondrial function to sustain cellular energy supply under hypoxic conditions, highlighting the critical role of the chitosan–phosphate crosslinked network in maintaining salidroside bioactivity. At the molecular signaling level, HIF-1α (Fig. 8D) and its downstream effectors EPO (Fig. 8E) and ET-1 (Fig. 8F) exhibited consistent expression trends. The high HIF-1α expression in the No-added model group represented passive compensation under severe hypoxia, often accompanied by functional impairment. The H-CH-TSP-UL group significantly downregulated HIF-1α levels, indicating that salidroside did not simply inhibit this pathway but rather improved mitochondrial respiratory efficiency and oxygen utilization, fundamentally alleviating cellular hypoxic perception and eliminating the dependence on high HIF-1α levels for survival (Guo et al., 2020). The consequent reductions in EPO and ET-1 levels respectively suggested that salidroside replaced the pro-survival role of EPO to directly protect cells from apoptosis, while suppressing hypoxia-induced vasoconstriction and inflammatory cascade reactions. In summary, ultrasound-assisted highly crosslinked nanoemulsions construct stable micellar carriers that enable precise modulation of salidroside release kinetics (Fig. 8G), allowing the salidroside to exert optimal anti-hypoxic and anti-inflammatory efficacy within cells.

## 4 Conclusion

In this study, we employed an ultrasound-driven strategy to construct a gel-like shell structure on the surface of nanoemulsions through the interfacial ionic crosslinking of chitosan and phosphate, for the efficient delivery of salidroside. We demonstrated that this ultrasound-mediated process facilitated the formation of a compact, uniform shell on the nanoemulsion interface. In particular, the H-CH-TSP-UL nanoemulsion achieved a crosslinking degree of 0.78, an encapsulation efficiency of 69.21%, and the slowest cumulative release profile, while exhibiting superior stability against pH fluctuations, ionic strength variations, enzymatic degradation, and long-term storage. In vitro simulated gastrointestinal digestion studies confirmed that this ultrasound-optimized crosslinked network achieved a salidroside bioaccessibility of 25% and promoted the formation of bioavailable mixed micelles. Notably, the released salidroside-loaded micelles significantly mitigated hypoxia-induced oxidative stress in RAW264.7 macrophages, downregulated HIF-1α signaling pathways, and restored impaired mitochondrial energy metabolism. This research will provide technical support for ultrasound as a carrier for nutrients in shell nucleus nanoemulsions.This study has established a non-thermal processing strategy for the construction of core-shell nanoemulsions via ultrasonic-driven interfacial ionic cross-linking, providing new insights into the design of functional nutrient delivery carriers. The proposed strategy is not only suitable for the stable encapsulation of heat-sensitive active ingredients such as rhodiolin, but also has the potential to be extended to oral delivery systems for a variety of natural bioactive compounds, offering a new technical approach to enhancing their bioavailability and bioactivity.

## CRediT authorship contribution statement

**Liang Dong:** Writing – original draft, Methodology, Formal analysis, Conceptualization. **Guicheng Liu:** Resources, Data curation. **Xin Zhang:** Resources, Data curation. **Xiaojin Zhang:** Validation, Methodology, Investigation. **Sijing Liu:** Validation, Methodology, Investigation. **Shuqi Niu:** Resources, Data curation. **Degui Chang:** Supervision, Resources. **Xujun Yu:** Supervision, Resources. **Jinlin Guo:** Supervision, Resources.

## Declaration of competing interest

The authors declare that they have no known competing financial interests or personal relationships that could have appeared to influence the work reported in this paper.

## Data Availability

Data will be made available on request.
